# Synovitis, Acne, Pustulosis, Hyperostosis, Osteitis (SAPHO) Syndrome Mimicking Bone Metastases in the Spine: A Presentation of Two Cases and Literature Review

**DOI:** 10.7759/cureus.64974

**Published:** 2024-07-20

**Authors:** Fernando Luiz Rolemberg Dantas, François Dantas, Rômulo Tscherbakowski Nunes de Guimarães Mourão, Bárbara Campos Mattos, Victor Kelles Tupy da Fonseca

**Affiliations:** 1 Neurological Surgery, Biocor Instituto, Belo Horizonte, BRA; 2 Neurological Surgery, Faculdade Ciências Médicas de Minas Gerais, Belo Horizonte, BRA

**Keywords:** bone, metastases, spine, syndrome, sapho

## Abstract

Synovitis, acne, pustulosis, hyperostosis, osteitis (SAPHO) syndrome is a rare disorder of unknown etiology with heterogeneous clinical manifestations. We describe two cases of patients diagnosed with SAPHO syndrome mimicking spinal bone metastases. A literature review was conducted to identify similar previously reported cases. The first patient was a 56-year-old woman with progressive back pain for six months who was referred to the neurosurgery department for suspected spinal metastases. A spinal CT scan revealed hyperdense lesions at T10 and hyperdense changes in the lumbar vertebrae. Spinal MRI demonstrated bone marrow edema that was hypointense on T1-weighted imaging and hyperintense on T2-weighted imaging in multiple thoracic vertebrae, and the PET/CT showed multiple skeletal lesions affecting the spine with low-to-moderate ^18^F-FDG uptake. Scintigraphy showed the characteristic “bull’s head” sign with increased uptake in the manubrium and bilateral sternoclavicular joints. The second patient was a 66-year-old woman with a four-month history of back pain, who was admitted with multiple spinal lesions. The diagnosis was made after bone scintigraphy demonstrated the characteristic findings of the syndrome. Both patients lacked cutaneous lesions on presentation but reported previous skin lesions. SAPHO syndrome is a rare condition, and bone lesions associated with the disease may be misdiagnosed as bone metastases. Knowledge of the syndrome and its imaging findings is essential for accurate diagnosis and treatment.

## Introduction

Synovitis, acne, pustulosis, hyperostosis, osteitis (SAPHO) syndrome is a rare condition that affects the skin, bones, and joints. Chamot et al. were the first to describe the clinical and radiological findings of this syndrome, which include synovitis, acne, pustulosis (often on the palms and soles), hyperostosis, and osteitis [1]. The most common clinical features of the SAPHO syndrome in adults are anterior chest wall pain, swelling, and tenderness. When the clinical presentation does not fit the typical disease pattern, it is difficult to distinguish SAPHO from other diagnoses, particularly bone tumors [2-10]. We describe two cases of SAPHO syndrome mimicking spinal metastases diagnosed at a single tertiary Brazilian institution. A literature review was conducted in Medline and Google Scholar using the terms “SAPHO,” “spine,” and “metastases” to identify similar cases previously reported. Articles published between January 1980 and May 2023 were reviewed.

## Case presentation

Case 1

**Figure 1:**
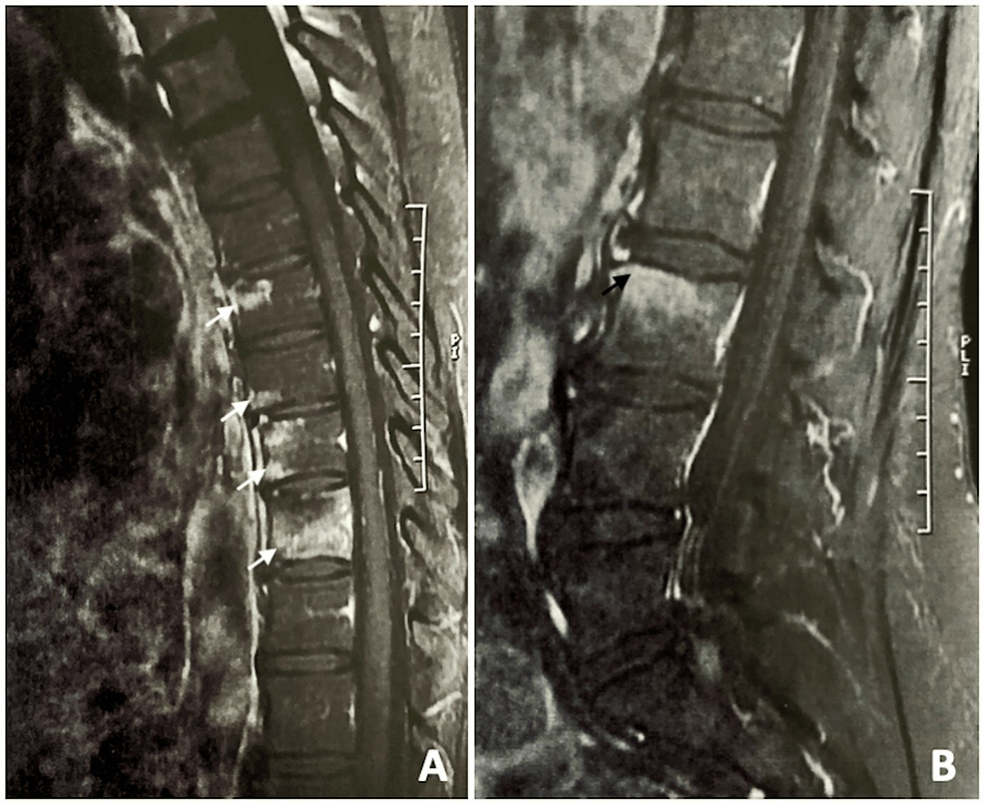
Gadolinium-enhanced sagittal T1-weighted MRI revealing contrast enhancement at the vertebral bodies of T7, T8, T9, T10 (white arrows) (A), and L3 (black arrow) (B).

A 56-year-old woman with a six-month history of back pain was referred to our institution for suspected bone metastases. She reported progressively worsening mechanical back pain. There was no history of weight loss, fever, or trauma, and she had no neurological deficits. Spinal MRI revealed multiple lesions in the vertebral bodies of T7-10 and L3, which were hypointense on T1 and hyperintense on T2/STIR, with contrast uptake, and ill-defined (Figure 1).

**Figure 2:**
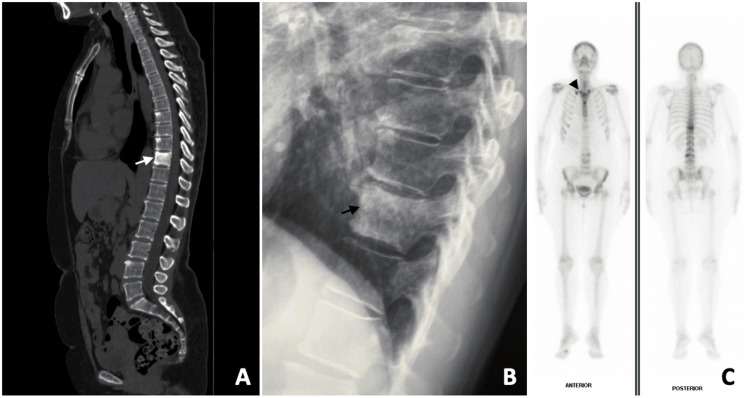
Sagittal view of a CT scan showing an ivory vertebra at T10 (white arrow) and multiple hyperdense lesions in the thoracic and lumbar spine (A). Lateral radiography of the thoracic spine showing the aspect of an ivory vertebra on T10 (black arrow) (B). Anterior and posterior views of whole-body bone scintigraphy with 99mTc-methylene diphosphonate. Increased tracer uptake was observed in both sternoclavicular joints and the sternum (“bull’s head” sign) (black arrowhead), as well as in the upper thoracic and lumbar vertebrae (C).

CT revealed sclerotic changes in the vertebral bodies of T7-10, L3, and L4 (Figure 2). As metastatic lesions were suspected and a primary tumor had not been identified, she underwent an L3 vertebral body biopsy, which revealed inflammatory changes and no signs of malignancy. Bone scintigraphy with 99mTc-methylene diphosphonate showed accumulation of tracer in the thoracic and lumbar spines, as well as increased tracer uptake in both sternoclavicular joints (“bull’s head” sign) (Figure 2).

**Figure 3:**
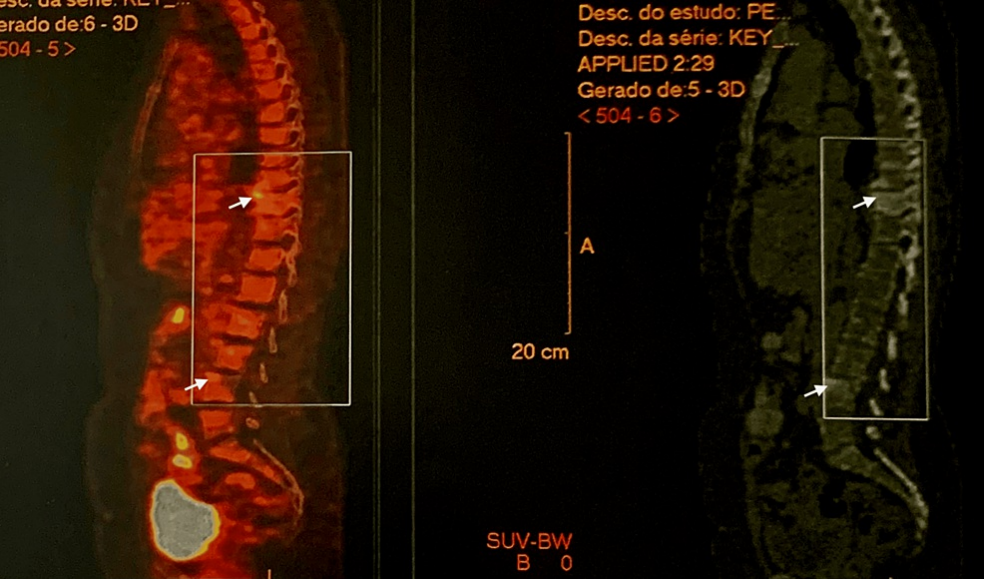
PET/CT scan showing multiple skeletal lesions affecting the spine with low to moderate 18F-FDG uptake (white arrows).

PET/CT showed contrast uptake in the vertebral bodies and the sternoclavicular joints (Figure 3).

**Figure 4:**
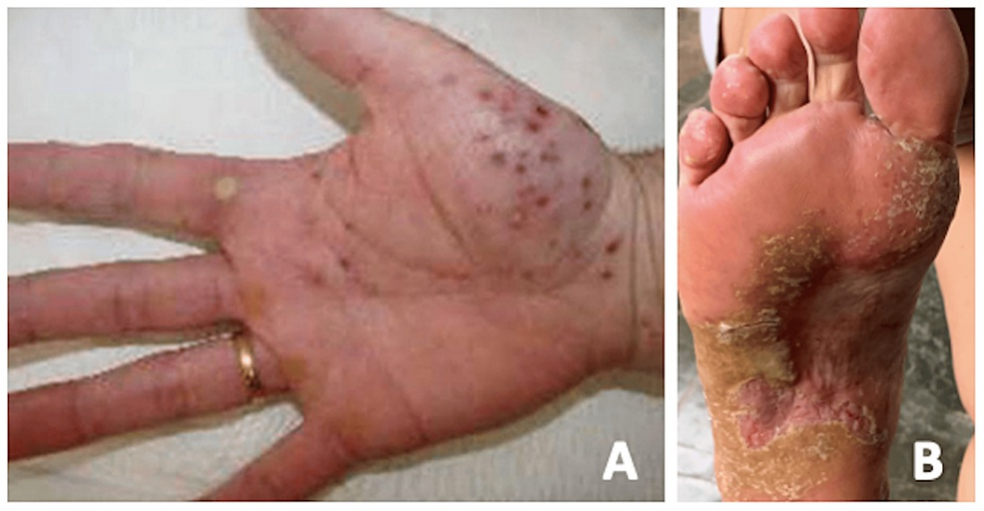
Photograph of the patient’s foot and hand demonstrating plantar (A) and palmar (B) pustulosis two years before hospital admission.

At the time of admission, she had no significant skin lesions, but on further questioning, the patient revealed that she had developed palmar and plantar pustulosis two years earlier (Figure 4). After reviewing her pictures showing the skin lesions and imaging, the diagnosis of SAPHO syndrome was suggested, and the patient was referred to the rheumatology department. The patient was administered methotrexate, prednisone, folic acid, and risedronate. The patient progressed well, with improvement in lower back pain and no recurrence of skin lesions at the two-year follow-up.

Case 2

**Figure 5:**
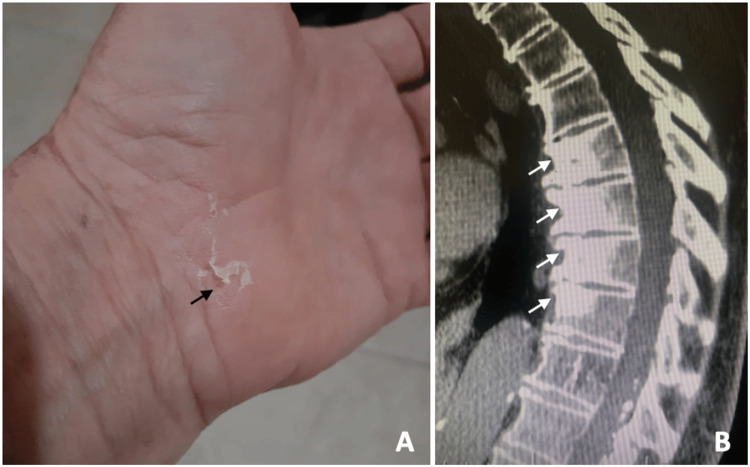
Photograph of the patient's hand demonstrating scaly phase (black arrow) (A). Sagittal CT scan showing marked sclerosis and subcortical bone irregularity, notably in the anterior half of both endplates of the T7, T8, and T9 vertebral bodies, as well as in the superior endplate of T10 (white arrows) (B).

A 66-year-old woman was referred to our institution with suspected spinal bone metastasis. She reported a history of back pain for four months. The neurological examination was unremarkable. On admission, the patient had no significant skin lesions (Figure 5).

**Figure 6:**
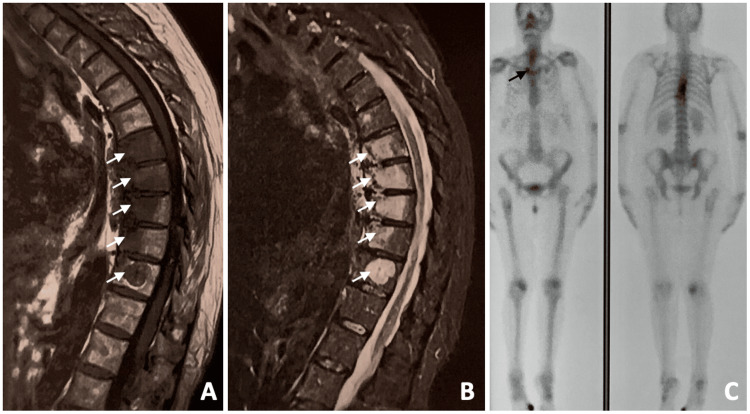
Sagittal T1-weighted (A) and T2-weighted (B) MRI showing signal abnormalities in multiple thoracic vertebral bodies (white arrows). Whole-body bone scintigraphy showing increased tracer uptake in the sternum, right sternoclavicular joint (black arrow), and thoracic spine (C).

Spinal CT revealed hyperdense lesions in the vertebral bodies at T7-T10 (Figure 5). Spinal MRI showed hypointense lesions on T1 and hyperintense lesions on T2-weighted images (Figure 6). Bone scintigraphy with 99mTc-MDP demonstrated uptake in the vertebral bodies from T8 to T10 and sternoclavicular joints (Figure 6).

A diagnosis of SAPHO was established, and the patient was referred for rheumatological evaluation and treatment. After two years of follow-up, there was no recurrence of back pain or skin lesions.

## Discussion

SAPHO syndrome was first described in 1987 by Chamot et al. and involves a rare group of inflammatory osteoarticular disorders commonly associated with skin disorders [1]. It is a rare condition, with an estimated prevalence of 1:10,000. However, its prevalence is thought to have been underestimated [11]. SAPHO syndrome may present at any age, although it tends to be most common in children and young to middle-aged adults [12]. The clinical presentation of SAPHO syndrome is heterogeneous, so patients may need to be examined by different experts to reach a comprehensive diagnosis [5].

The diagnostic criteria proposed by Benhamou et al. include (1) osteoarticular manifestations of acne conglobata, acne fulminans, or hidradenitis suppurativa; (2) osteoarticular manifestations of palmar-plantar pustulosis; (3) hyperostosis involving the anterior chest wall, spine, or limbs with or without dermatosis; and (4) chronic recurrent multifocal osteomyelitis with or without dermatosis [13]. Diagnosis of the syndrome involves the exclusion of other diseases such as septic osteomyelitis, infectious chest wall arthritis, infectious palmoplantar pustulosis, diffuse idiopathic skeletal hyperostosis, palmoplantar keratoderma, and osteoarticular manifestations of retinoid therapy [13].

Skin involvement may be transient or may develop decades after the onset of bone changes and is not considered essential for diagnosis. At least 15% of adult patients never have skin manifestations [3]. Patients can present with sternocostal-clavicular hyperostosis and/or hyperostosis of the spine, pelvis, or peripheral skeleton in the absence of palmoplantar pustulosis [10]. Sacroiliitis associated with SAPHO syndrome occurs in 13-52% of cases and is often unilateral. HLA-B27 has been reported to be positive in 13-30% of patients [3,11].

Several forms of spinal involvement in SAPHO syndrome have already been described, including spondylodiscitis [14], vertebral fracture and spinal cord injury [15], pathological fractures [16], and isolated osteolytic lesions [17]. There have been reports of metastatic cancer mimicking SAPHO syndrome [12,18], SAPHO syndrome associated with neoplasia [19], and SAPHO syndrome diagnosed during cancer treatment [5].

Our cases were initially thought to have possible spinal metastases, and SAPHO was diagnosed only after an extensive workup, as none of the patients had active skin lesions. Only 11 cases, including ours, of SAPHO syndrome that were initially diagnosed as possible bone metastases to the spine have been reported in the literature (Table 1).

**Table 1:** Characteristics of the patients identified in the literature review SI, sacroiliac

Author and year	Sex	Age (years)	Presentation	Spine segment	Radiological images
Theumann et al. (2005) [10]	F	46	Previous breast cancer, palmar pustulosis, and sacroiliitis	Lumbar	X-ray, CT scan, and bone scintigraphy
Takeuchi et al. (2007) [9]	F	50	Back pain and neck pain	Cervical, thoracic, lumbar, and SI joint	X-ray, MRI, bone scintigraphy, and PET/CT
Inoue et al. (2007) [4]	F	74	Back pain	Thoracic, lumbar, and SI joint	X-ray, MRI, bone scintigraphy, and PET/CT
Patel et al. (2009) [7]	F	20	Back pain	Thoracic and SI joint	MRI, CT scan, bone scintigraphy, and PET/CT
Abuhid et al. (2010) [2]	F	44	Pain in the sternoclavicular and sacroiliac joints and plantar pustulosis	SI joint	X-ray, bone scintigraphy, and PET/CT
Mann et al. (2012) [6]	F	52	Back pain	Thoracic and lumbar	X-ray, CT scan, and bone scintigraphy
Kubo et al. (2021) [5]	F	69	Back pain and palmar pustulosis	Thoracic and lumbar	CT scan, MRI, and bone scintigraphy
Biuden et al. (2021) [3]	F	46	Neck pain	Cervical	X-ray, CT scan, MRI, and bone scintigraphy
	M	46	Back pain	Thoracic, lumbar, and SI joint	CT scan, MRI, bone scintigraphy
Our study	F	56	Back pain	Thoracic and lumbar	X-ray, CT scan, MRI, bone scintigraphy, and PET/CT
	F	66	Back pain	Thoracic	CT scan, MRI, bone scintigraphy, and PET/CT

Most patients were adults and women (10F/1M, range 20-74 years), and back pain was the most common clinical manifestation. In this review, most patients had a recent onset of back pain, ranging from two to eight months. Only three patients had a longstanding history of back pain (one, three, and 20 years). The thoracic and lumbar segments were the most affected, and the cervical spine was affected in only two cases. Among the previously reported cases, only two patients had palmoplantar pustular skin lesions on admission [2,5].

Several imaging tests were necessary to define the diagnosis in all cases, highlighting the difficulty of making an accurate diagnosis. The most commonly used tests were radiographs, CT scans, MRIs, bone scintigraphy, and PET/CT.

A study by Xu et al. compared the imaging values of 99mTc-MDP bone scans and 68Ga-FAPI-04 PET/CT. The authors analyzed images from 19 patients and concluded that PET/CT was more sensitive than bone scans for evaluating osteoarticular lesions in SAPHO syndrome and could detect early-stage bone and joint lesions [20]. Sun et al. analyzed bone scintigraphy and PET/CT in 26 patients and concluded that FDG PET/CT showed a comparable capacity to reveal skeletal lesions with bone scintigraphy [21]. Bone scintigraphy can be very useful, as it not only shows increased uptake in the affected sites but also reveals silent lesions. Anterior chest wall involvement is the most frequent finding, and the “bull’s head” sign (an increased uptake in the manubrium and bilateral sternoclavicular joints) is a characteristic feature of SAPHO syndrome. This can be seen in 10-31% of the cases [20].

Most patients with SAPHO syndrome and vertebral involvement have multilevel disease; the thoracic spine is the most commonly involved, followed by the lumbosacral spine. Contiguous involvement of ≥2 vertebral bodies is seen in most patients. The lesions are usually hypointense on T1-weighted images and hyperintense on T2-weighted images, and most lesions present with gadolinium enhancement [22].

The treatment options for SAPHO syndrome are heterogeneous and include nonsteroidal anti-inflammatory drugs, bisphosphonates, and conventional and biological disease-modifying antirheumatic drugs [23]. No consensus has been reached regarding the treatment of SAPHO syndrome, and biological drugs with specific molecular targets have been increasingly studied [24].

The prognosis of SAPHO syndrome is considered to be relatively good; however, recent studies have reported a high incidence of malignancy associated with SAPHO syndrome [25]. Despite the good overall prognosis in most patients, SAPHO appears to be a chronic disease with recurrent exacerbations and remissions, severely undermining patients’ quality of life [21].

## Conclusions

SAPHO syndrome should be suspected in patients with multifocal osteitis or arthritis, particularly when associated with skin manifestations. Combinations of multimodal imaging modalities are helpful for diagnosis. Adequate knowledge of the disease is essential for the correct distinction between SAPHO syndrome and bone metastasis.
